# Dedicated sellar magnetic resonance imaging protocols without contrast enhancement in girls with central precocious puberty: prevalence of pathologic lesions and clinical correlation

**DOI:** 10.20945/2359-3997000000412

**Published:** 2021-11-11

**Authors:** Sang Young Park, Na Yeong Lee, Min Ho Jung, Gye Yeon Lim

**Affiliations:** 1 The Catholic University of Korea College of Medicine Department of Radiology Seoul Korea Department of Radiology, Yeouido St. Mary's Hospital, College of Medicine, The Catholic University of Korea, Seoul, Republic of Korea; 2 The Catholic University of Korea College of Medicine Department of Pediatrics Seoul Korea Department of Pediatrics, Yeouido St. Mary’s Hospital, College of Medicine, The Catholic University of Korea, Seoul, Republic of Korea

**Keywords:** Girls, precocious puberty, brain, sellar, magnetic resonance imaging

## Abstract

**Objective:**

Contrast-enhanced brain magnetic resonance imaging (MRI) is routinely performed in children with central precocious puberty (CPP). We evaluated the value of a dedicated sellar MRI protocol without contrast enhancement in girls with CPP.

**Subjects and methods:**

This study included 261 girls diagnosed with CPP. We performed sellar MRI scanning without gadolinium enhancement of the hypothalamic-pituitary area (HPA) at the pituitary level, including additional T2-weighted imaging of whole-brain scans to check for other lesions. We evaluated the prevalence of intracranial lesions via this MR protocol. In addition,the correlation between the clinical parameters and morphology of the pituitary gland on the images was assessed.

**Results:**

Intracranial lesions were detected in 17 (6.5%) of the 261 girls. Of the 17 girls with abnormalities, 16 (94.1%) had findings in brain areas other than the HPA. The weight, height, Tanner stage of patients were significantly (p < 0.05) higher in the group with greater pituitary height. Patient weight and height, Tanner stage of breast development, and luteinizing hormone (LH) levels were significantly (p < 0.05) greater in those with a higher pituitary grade as determined on sellar MRI.

**Conclusion:**

A dedicated unenhanced sellar MRI protocol provides valuable information on brain lesions and pituitary morphology. We found a significantly low prevalence of brain lesions among girls with CPP. Analysis of the height or shape of the pituitary gland on sellar MRI revealed significant correlations with the weight, height, Tanner stage, and LH levels of the patients.

## INTRODUCTION

Central precocious puberty (CPP) is early development of secondary sexual characteristics before the age of 8 years in girls, caused by premature activation of the hypothalamic-pituitary-gonadal axis (HPA) (1). Once CPP is confirmed by laboratory testing, central nervous system (CNS) imaging is performed to identify possible CNS abnormalities (2-5). The prevalence of CNS abnormalities in girls is low, at 0%-27%, and decreases with increasing age (6-8). Recently, there has been controversy has arisen over whether cranial imaging is necessary for all girls with CPP (9). Some pediatric endocrinologists recommend that girls with CPP undergo cranial magnetic resonance imaging (MRI) only if they are < 6 years of age (3). Some doctors and parents become concerned, however, if CNS abnormalities are not ruled out.

Contrast-enhanced brain MRI (CE-MRI) is the diagnostic modality that is used to exclude organic causes of CPP. However, CE-MRI contrast injection that is part of CEMRI, has several disadvantages, including discomfort to the patient, the need for sedation, and the high cost of the procedure (10). In addition, the clinical impact and toxicity of gadolinium-based contrast agent agents is unclear, and long-term safety has not been fully established in pediatric patients (11,12). In consideration of these issues, guidelines for CPP screening tests need refinement. Therefore, we investigated the value of a dedicated sellar MRI protocol to evaluate the hypothalamic-pituitary axis (HPA), including additional T2-weighted imaging (T2WI) of the entire brain without contrast enhancement, in girls with CPP, and we discuss the clinical findings that justify its use. The primary aim of this study was to evaluate the prevalence and types of intracranial lesions in girls with CPP by age groups. A second aim was to evaluate the correlations of clinical and biochemical characteristics according to the morphology (height and shape) of the pituitary gland in sellar MRI images.

## SUBJECTS AND METHODS

### Patients

We retrospectively reviewed the records of girls diagnosed with CPP who underwent unenhanced sellar MRI at our hospital between January 2011 and July 2020. Patients with missing clinical data were excluded from the study. In total, 261 girls were included in the final analysis. The study protocol was approved by the institutional review board of our committee (SC21RISI0028). The requirement for informed consent was waived due to the retrospective nature of the study. The research was conducted in accordance with principles of the Declaration of Helsinki.

### Clinical features and CPP diagnosis

The CPP diagnosis was based on clinical and biochemical data. The clinical characteristics included age at diagnosis, age at the time of the MRI, age at thelarche/pubarche, weight (kg), height (cm), body mass index (BMI), mid-parental height, and the Tanner stage of breast and pubic hair development, as assessed by a pediatric endocrinologist. Standard deviation scores (SDSs) for height, weight, and BMI were calculated using 2017 National Growth Charts. The mid-parental height was calculated as (mother’s height + (father’s height − 13)/2 and denoted as SDS. Bone age (BA) was assessed based on the match between a left hand X-ray image and the Greulich and Pyle atlas. The gonadotropin-releasing hormone (GnRH) stimulation test, which is the gold standard for diagnosing CPP, was performed to obtain biochemical data. Serum levels of luteinizing hormone (LH) and follicle-stimulating hormone (FSH) were measured before and 30 min after intravenous (IV) injection of GnRH (100 µg Relefact; Sanofi-Aventis, Frankfurt am Main, Germany). CPP was diagnosed after it was confirmed that the patient had developed breasts before the age of 8 years, and had an advanced BA > 2 standard deviations (SDs) above the patient’s chronological age. The criterion for a CPP diagnosis was defined as a peak LH level ≥ 5 mIU/mL after a GnRH stimulation test. Serum LH and FSH levels were measured using immunoradiometric assays (BioSource, Nivelles, Belgium).

### Sellar MRI protocol and image interpretation

**Figure 1:**
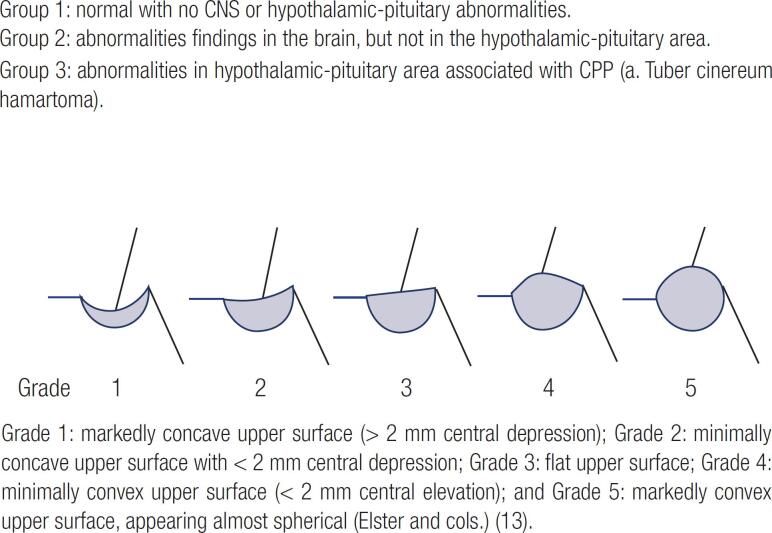
Reconstructed scheme for grading the pituitary gland shape.

We used a Signa Excite 1.5 T MRI scanner (GE Healthcare, Milwaukee, WI, USA) before 2016 and a Skyra 3 T MRI scanner (Siemens Healthcare, Erlangen, Germany) after 2016. MRI scans of the HPA were obtained in the coronal, sagittal, and axial planes at the pituitary level. The sagittal plane views were positioned parallel to the midline of the pilot scan in the coronal plane. We also performed a whole-brain axial T2WI scans to check for lesions. The imaging sequences and parameters are reported in Supplementary Table 1. Contrast enhancement was not used for the initial examinations in any of the patients. Only five of the 17 patients in whom abnormalities were found by initial MRI underwent additional contrast-enhanced scans. The MRI images of all patients were analyzed and interpreted by two experienced radiologists (SA Park and GY Lim). Pituitary shape, height, and stalk, and the presence of other abnormities in the HPA were verified. The mid-sagittal section was defined by visualizing the anterior and posterior pituitary lobes, along with the pituitary stalk, in the same slice. The height of the pituitary gland was measured at the site of insertion of the stalk as the maximum vertical distance between the upper and the lower borders of the gland. In the present study, increased pituitary height (pituitary hyperplasia) was considered as a height greater than 6 mm (13). The shape of the pituitary gland on sagittal images was graded using the scoring system of Elster and cols. (13) (Figure 1). In this system, the grades were as follows: 1, markedly concave upper surface (> 2 mm central depression); 2, minimally concave upper surface with < 2 mm central depression; 3, flat upper surface; 4, minimally convex upper surface (<2 mm central elevation); and 5, markedly convex upper surface, appearing almost spherical. The pooled prevalence of intracranial lesions was calculated, and stratified by age group (< 6, 6-8, and > 8 years). We also assessed changes in the clinical and laboratory parameters according to the height and shape of the pituitary gland in the images.

### Statistical analyses

Statistical analyses were performed using IBM SPSS Statistics software (version 26.0; IBM Corp., Armonk, NY, USA). Data are expressed as the mean ± SD. The Mann-Whitney *U* test was used to compare the MRI findings and clinical parameters between the groups. The Jonckheere-Terpstra test was used to identify trends in the clinical characteristics according to pituitary shape. In all analyses, p < 0.05 was taken to indicate statistical significance.

## RESULTS

### Prevalence of intracranial lesions in MRI images according to age group

In our cohort, 0.8% (n = 2) of the girls were aged < 6 years, 46% (n = 120) were aged 6-8 years, and 53.2% (n = 139) were aged 8-9 years (Table 1). The patients were divided into the following three groups according to the MRI findings: group 1, normal with no CNS or HPA abnormalities (n = 244, 93.5%); group 2, abnormalities in the brain region but not in the HPA region: pineal cysts (n = 12), choroidal fissure cysts (n = 2), sphenoid sinus retention cysts (n = 1), and neuroepithelial cysts (n = 1) (group 2, n = 16, 6.1%); and group 3, pathological abnormalities in the HPA associated with CPP (n = 1, 0.4%). The single patient in group 3 was a 4-year-old girl with a hypothalamic hamartoma (Table 1). Group 3 abnormal findings were detected in one of two girls aged < 6 years, whereas there were no abnormal findings in the girls with CPP aged 6-8 years or > 8 years. Only 6 of 120 girls (2.3%) aged 6-8 years and 10 of 139 girls (3.8%) with CPP aged > 8 years had group 2 abnormal findings (Table 1).

**Table 1:** MR characteristics of the patients according to age

MR Characteristics	Age < 6 (n = 2)	6 ≤ Age <8 (n = 120)	8 ≤ Age (n = 139)	Total
Group 1	1 (0.4%)	114 (43.7%)	129 (49.4%)	244 (93.5%)
Group 2	0 (0.0%)	6 (2.3%)	10 (3.8%)	16 (6.1%)
Group 3	1^a^ (0.4%)	0 (0.0%)	0 (0.0%)	1 (0.4%)
Total	2 (0.8%)	120 (46.0%)	139 (53.2%)	261 (100%)

Group 1: normal with no CNS or hypothalamic-pituitary abnormalities.

Group 2: abnormalities findings in the brain, but not in the hypothalamic-pituitary area.

Group 3: abnormalities in hypothalamic-pituitary area associated with CPP (a. Tuber cinereum hamartoma).

For all five patients who underwent a subsequent contrast-enhanced MRI scan, there were no additional diagnostic findings in the enhanced images compared to the non-enhanced ones.

### Clinical characteristics according to pituitary height

The clinical characteristics of the patients are summarized in Supplementary Table 2. Clinical characteristics according to pituitary height are presented in Table 2. Patients with increased pituitary height were significantly (p < 0.05) heavier, taller, and at a later Tanner stage than those with normal pituitary height (Table 2). No significant differences in mid-parental height or other clinical features were observed between the two groups (Table 2). BMI (SDS) was 0. 73 ± 1.91 in the pituitary hyperplasia group and 0.18 ± 0.92 in the group with normal pituitary height (p = 0.12).

**Table 2:** Clinical characteristics according to pituitary height

	Total (n = 261)	Normal pituitary height (< 6 mm) (n = 208)	Increased pituitary height (≥ 6 mm) (n = 53)	P-value
Age at diagnosis (m)	95.82 ± 9.30	95.65 ± 8.85	96.49 ± 10.95	0.326
Age at time of MR (m)	97.21 ± 9.34	97.06 ± 8.84	97.83 ± 11.19	0.333
Age at thelarche/pubarche (m)	91.33 ± 9.50	91.30 ± 9.38	91.43 ± 10.06	0.747
Weight_dx (SDS)	0.61 ± 0.97	0.49 ± 0.83	1.04 ± 1.31	0.006*
Height_dx (SDS)	0.81 ± 0.87	0.74 ± 0.88	1.07 ± 0.78	0.008*
BMI_dx (SDS)	0.29 ± 1.21	0.18 ± 0.92	0.73 ± 1.91	0.123
Mid-parental height (SDS)	-0.18 ± 0.73	-0.21 ± 0.73	0.10 ± 0.73	0.355
Tanner stage- breast	2.50 ± 0.55 2.5	2.43 ± 0.52 2.5	2.76 ± 0.60 3.0	0.000*
Tanner stage- pubic hair	1.04 ± 0.25 1.0	1.02 ± 0.17 1.0	1.11 ± 0.42 1.0	0.014*
BA-CA (m)	21.62 ± 9.88	21.14 ± 9.79	23.51 ± 10.12	0.091
Basal LH (IU/L)	0.52 ± 1.03	0.46 ± 0.79	0.79 ± 1.67	0.164
Peak LH (IU/L)	16.92 ± 16.12	14.90 ± 12.03	24.88 ± 25.36	0.388
Basal FSH (IU/L)	2.19 ± 1.20	2.10 ± 1.03	2.57 ± 1.67	0.147
Peak FSH (IU/L)	13.80 ± 5.84	13.46 ± 4.96	15.15 ± 8.37	0.267

SDS: standard deviation score; m: months; BA-CA: bone age-chronological age; Weight/Height/BMI_Dx: Weight/Height/BMI at the age of diagnosis; Mid-parental height: ((Height of Father – 13) + Height of Mother) / 2; LH, luteinizing hormone; FSH: follicle-stimulating hormone.

* p<0.05.

### Trends in clinical characteristics according to pituitary shape

The trends in the clinical characteristics according to pituitary shape are shown in Table 3. Patient weight, height, BMI, Tanner stage of breast development, and baseline/peak LH ratio were significantly greater in those with a higher pituitary grade as determined on sellar MRI. The obesity rate increased with pituitary shape maturation (p = 0.049). No significant increasing or decreasing trends were observed in mid-parental height (p = 0.60) or other parameters.

**Table 3:** Clinical characteristics according to pituitary shape

	Pituitary shape grade						
	1 (n = 2)	2 (n = 51)	3 (n = 124)	4 (n = 63)	5 (n = 21)	Std. J-T statistic	P-value
Age at diagnosis (m)	94.50 ± 0.71	95.18 ± 8.90	96.22 ± 8.97	94.92 ± 9.12	97.86 ± 12.78	0.637	0.524
Age at time of MR (m)	95.50 ± 0.71	96.53 ± 8.83	97.60 ± 8.97	96.37 ± 9.11	99.29 ± 13.33	0.710	0.477
Age at thelarche/pubarche (m)	90.00 ± 4.24	91.18 ± 8.97	91.49 ± 9.93	91.08 ± 8.24	91.57 ± 12.45	0.317	0.751
Weight_Dx (SDS)	0.19 ± 0.55	0.42 ± 0.77	0.46 ± 0.84	1.00 ± 1.24	0.74 ± 0.92	2.931	0.003*
Height_Dx (SDS)	0.56 ± 0.11	0.69 ± 0.86	0.72 ± 0.89	1.00 ± 0.81	1.07 ± 0.83	2.739	0.006*
BMI_Dx (SDS)	-0.14 ± 0.88	0.09 ± 0.87	0.15 ± 0.90	0.72 ± 1.82	0.32 ± 1.00	1.967	0.049*
Mid-parental height (SDS)	-0.54 ± 1.03	-0.23 ± 0.61	-0.18 ± 0.79	-0.15 ± 0.72	-0.19 ± 0.74	0.528	0.598
Tanner stage-breast	2.75 ± 0.35 2.75	2.33 ± 0.52 2.50	2.45 ± 0.50 2.50	2.60 ± 0.62 2.50	2.86 ± 0.50 3.00	3.396	0.001*
Tanner stage-pubic hair	1.00 ± 0.00 1.00	1.02 ± 0.14 1.00	1.02 ± 0.18 1.00	1.08 ± 0.37 1.00	1.10 ± 0.30 1.00	1.923	0.055
BA-CA (m)	12.50 ± 0.71	20.81 ± 7.61	21.41 ± 10.93	21.82 ± 8.62	25.11 ± 11.67	1.653	0.098
Basal LH (IU/L)	3.52 ± 4.36	0.29 ± 0.22	0.38 ± 0.52	0.91 ± 1.72	0.56 ± 0.61	2.764	0.006*
Peak LH (IU/L)	11.76 ± 5.66	12.91 ± 9.12	14.90 ± 11.19	21.07 ± 22.76	26.71 ± 23.90	2.099	0.036*
Basal FSH (IU/L)	2.67 ± 1.65	2.03 ± 0.88	2.15 ± 1.02	2.04 ± 1.17	3.27 ± 2.13	0.684	0.494
Peak FSH (IU/L)	12.19 ± 6.58	13.94 ± 4.88	13.50 ± 4.89	13.98 ± 7.77	14.80 ± 6.76	-0.357	0.721

Std. J-T statistic: standard Jonckheere-Terpstra statistic; SDS: standard deviation score; m: months; BA-CA: bone age-chronological age; Weight/Height/BMI_Dx: Weight/Height/BMI at the age of diagnosis; Mid-parental height: ((Height of Father – 13) + Height of Mother) / 2; LH: luteinizing hormone; FSH: follicle-stimulating hormone.

* p<0.05.

## DISCUSSION

CPP can be idiopathic or associated with CNS abnormalities, such as hamartomas or brain tumors. The prevalence of CNS abnormalities is much lower in girls (0%-27%) than in boys, and decreases with age (6-8). Recent studies that have classified girls with CPP according to age at diagnosis reported that 17.1%-26.9% of girls diagnosed at age < 6 years have a CNS pathology compared to 0%-1.9% of those diagnosed at age ≥ 6 years (9,14-17). In our study, abnormal findings occurred in 6.5% of the cases, but most (6.1%) were in the brain regions outside the HPA, and none of the girls aged > 6 years had lesions in the HPA region related to CPP. However, these results may have been influenced by selection bias. The reason for this feature may be that 53.2% of the patients were aged 8-9 years. Generally, routine sellar MRI was performed for girls with CPP who were younger than 6-8 years. In our study, MRI was performed in girls aged 8-9 years to evaluate the patients at the time of the diagnosis. The precise onset of secondary sexual characteristic development was unknown because the parents did not know the exact onset age. Therefore, the onset of secondary sexual characteristic development could have occurred at ages younger than those we identified. Thus, there could have been considerable gap between patient onset age and the age at the time of diagnosis. Nevertheless, some cases showed early puberty in this age group. In these cases, we explained the relationships between brain lesions and CPP to the patients’ parents, and performed MRI on request.

There is debate whether such cranial imaging is necessary for all girls with CPP (14-17). Some pediatric endocrinologists recommend that girls with CPP only undergo cranial MRI if they are < 6 years old (9). Some doctors and parents become concerned when girls with CPP forgo exams. Contrast-enhanced MRI is routinely performed in many tertiary care centers to rule out brain abnormalities in girls diagnosed with CPP, despite the disadvantages of administering contrast agents and IV sedation, which is often needed in young children (10-12). The standard sellar MRI protocol includes 2-3 mm thick, T1- and T2-weighted images in the sagittal and coronal planes. Conventional spin-echo techniques are usually employed for T1-weighted imaging, whereas turbo/fast spin-echo sequences are obtained for T2WI. Thin-slice (2-3 mm thick) contrast enhanced T1-weighted sequences should be obtained in the sagittal and coronal planes (18,19). Such protocols typically take 30-40 minutes and can be prolonged, especially in pediatric examinations. Therefore, the screening test should be more concise, and we evaluated the value of a dedicated sellar MRI protocol to evaluate the HPA and an additional T2WI of the entire brain without contrast enhancement in girls with CPP. This simplified dedicated protocol shortened the scanning time by 10-15 min, resulting in a reduction in anxiety of the parents and patients and lower costs, due to not performing sedation or using a contrast medium.

Because pituitary microadenoma and Rathke’s cleft cyst can only be seen in contrast-enhanced images, and the diagnosis of these lesions can be missed in non-contrast images. Nevertheless, the clinical significance of these lesions in girls with CPP has not been demonstrated. It would have been ideal to compare this proposed protocol with contrast enhancement methods to validate the protocol, but this would be ethically problematic and practically impossible. By contrast, comparing a historical cohort of CPP patients who underwent contrast-enhanced MRI with a group that did not undergo contrast-enhanced MRI is possible. These data would significantly increase the value of the study.

We could not rule out the possibility that some brain lesions were not found due to the lack of MRI contrast enhancement in the study protocol, but we were able to describe the morphometry of the pituitary gland and analyzed the correlations between pituitary morphology and the clinical parameters of the patients. The height and shape of the pituitary gland varied among patients on sellar MRI. We were interested in determining whether there were correlations with pituitary height and shape. Patients with an increased pituitary height were significantly heavier, taller, and at a later Tanner stage than those with normal pituitary height. No significant difference in mid-parental height was observed between the two groups. Among the other laboratory parameters, basal and peak LH levels were significantly higher in the pituitary hyperplasia group. The pituitary height cutoff standard in our study referred to the pituitary hyperplasia standard in previous studies. In the future, it will be necessary to establish objective standards by comparing a normal control group of the same age.

In addition, patient weight, height, BMI, Tanner stage of breast development, and the baseline/peak LH ratio were significantly greater in those with a larger pituitary grade as determined by sellar MRI. In other words, the values of the clinical parameters tended to be increased significantly in accordance with the convex nature of the pituitary gland’s upper margin. No significant increasing or decreasing trends were observed for mid-parental height.

BMI (SDS) tended to increase with maturation of the pituitary shape (p = 0.049).

The present study had several limitations. First, our patients only underwent unenhanced MRI, so we could not compare the diagnostic utility between unenhanced and contrast-enhanced MRI results. Second, the proportion of patients aged > 8 years was larger than in previous studies; this may have affected the results. Third, a single-center study is subject to a number of biases, including selection bias, which may limit the generalizability of the findings; however, the results are meaningful due to the large sample size.

In conclusion, unenhanced dedicated sellar MRI of the HPA, with additional T2WI of the brain region, provided valuable information to detect organic lesions and determine pituitary gland morphology. We observed a significantly low prevalence of brain lesions among girls with CPP. As this was only a non-contrast study, comparative studies with contrast-enhanced MRI are required to validate the proposed protocol. Analysis of the correlations between the clinical characteristics and sellar MRI findings indicated that patient weight, height, BMI, Tanner stage, and LH level were associated with pituitary height or shape on sellar MRI. The results of this study suggest that clinicians should carefully examine the morphology of the pituitary gland in addition to determination of pathological causes of CPP on MRI in these patients.

## Appendix

**Supplemental Table 1:** Sellar MR sequences and parameters

Sequence	TR(ms)	TE(ms)	FOV (mm)	Matrix size	Slice thickness (mm)	Slice gap (mm)	Flip angle
**1.5T (Signa Excite)**							
T2W FSE AX	4000	107	220x220	320x224	5	7	90
T2W FRFSE COR	4000	105.6	180x180	320x256	3	4	90
T1W FSE AX	500	18.7	160x160	320x224	3	4	90
T1W FSE COR	500	18.7	180x180	320x224	3	4	90
T1W TSE SAG	500	12.5	180x180	320x224	3	4	90
**3T (Skyra)**							
T2W TSE TRA AX	6660	100	180x200	512x325	4	4.4	150
T2W TSE COR	5190	116	160x160	384x269	1.5	1.5	150
T1W SE TRA AX	500	9.6	180x180	320x192	1.5	1.5	150
T1W SE COR	500	9.6	160x160	320x192	1.5	1.5	150
T1W SE SAG	500	9.6	160x160	320x192	1.5	1.65	150

**Supplementary Table 2:**

No.	DOB	MR date	Age at MR (m)	Age at Dx (m)	Age of thelache /puberache (m)	bone age (m)	Pit_height (mm)	Pit_shape	Pit_hyperplasia	BA-CA (m)	Height(cm)	Weight(kg)	H_Dx(SDS)	W_Dx(SDS)	BMI_Dx(SDS)	H_MR(SDS)	W_MR(SDS)	BMI_MR(SDS)	MPH(cm)	MPH_SDS(19yr)	Tstage_Br	Tstage_Pu	Basal LH (IU/L)	Peak LH (IU/L)	Basal FSH (IU/L)	Peak FSH (IU/L)	MR findings
1	2004-04-19	2008-08-11	51	51	43	86,00	7,4	5	2	38,00	110,8	23,8	1,710	2,750	2,651	2,099	3,123	2,803	164,5	0,69	3,5	1,0	1,37	61,80	7,66	33,50	Tuber cinereum hamartoma
2	2014-02-20	2020-07-17	76	74	74	114,00	4,0	2	1	38,00	124,0	20,4	1,782	-0,248	-2,011	1,546	-0,395	-2,007	161,3	0,05	2,0	1,0	0,30	5,53	1,90	17,30	Noncomplicated small arachnoid cyst, posterior fossa
3	2014-02-04	2020-07-15	77	76	74	114,00	4,8	2	1	37,00	131,2	28,0	3,032	1,670	0,233	2,918	1,601	0,216	167,0	1,17	2,5	1,0	0,20	10,26	0,65	5,86	Noncomplicated small arachnoid cyst, right temporal fossa
4	2009-03-07	2016-07-11	88	87	85	122,50	4,9	2	1	34,50	134,2	27,3	2,088	1,010	-0,060	2,200	0,694	-0,082	162,5	0,29	1,5	1,0	0,22	34,82	2,93	24,33	R/O retension cyst, Rt. sphenoid sinus. D/Dx mucocele
5	2005-03-15	2012-10-05	90	89	84	122,50	6,1	4	2	32,50	134,9	31,0	1,873	0,777	-0,265	2,113	1,275	-0,285	163,5	0,49	2,5	1,0	0,10	8,10	2,02	18,89	R/O neuroepithelial cyst
6	2012-09-10	2020-04-14	91	90	81	108,00	6,6	4	2	17,00	127,5	25,5	0,739	0,146	-0,368	0,639	0,077	-0,387	161,0	-0,01	2,5	1,0	0,20	5,68	1,63	12,49	R/O a small pineal cyst
7	2006-12-07	2014-11-27	95	95	92	122,50	6,0	4	2	27,50	136,0	30,0	1,798	0,776	-0,164	1,798	0,776	-0,164	165,5	0,88	2,0	1,0	0,20	5,27	1,05	17,46	A noncomplicated arachnoid cyst in the Rt middle cranial fossa
8	2006-08-04	2014-10-24	98	98	97	108,00	4,9	3	1	10,00	134,1	31,0	0,660	0,330	-0,004	1,177	0,764	-0,004	165,5	0,88	3,0	1,0	0,20	10,06	0,91	6,88	R/O Septo-optic dysplasia.
9	2011-03-03	2019-05-09	98	97	93	132,00	6,0	5	2	34,00	139,3	33,0	2,156	1,163	0,186	2,060	1,101	0,160	163,5	0,49	3,0	1,0	0,24	7,79	2,32	7,93	A choroidal fissure cyst, right
10	2012-03-18	2020-05-21	98	97	95	108,00	4,7	3	1	10,00	124,3	25,8	-0,557	-0,244	0,034	-0,651	-0,309	0,009	157,0	-0,84	3,0	1,0	0,20	28,64	1,94	11,43	R/O pineal cyst
11	2005-05-18	2013-09-21	100	99	96	132,00	6,5	5	2	32,00	132,2	29,0	1,849	1,995	1,687	0,655	0,265	1,659	162,0	0,19	3,0	2,0	0,20	14,50	1,29	10,01	Slightly prominent bilateral lateral ventricles and extraaxial CSF space
12	2011-11-16	2020-05-13	101	100	97	120,00	6,3	4	1	19,00	135,5	32,5	1,234	0,898	0,433	1,141	0,838	0,407	161,5	0,09	3,0	1,0	0,26	24,04	1,83	7,46	Mild widening of subarachnoid space of both frontoparietal region and prominent both lateral ventricles with uncertain cause
13	2011-06-30	2020-03-27	104	104	100	132,00	5,0	3	1	28,00	126,9	25,8	-0,700	-0,689	-0,474	-0,700	-0,689	-0,474	151,5	-2,02	2,5	1,0	0,57	5,17	4,84	9,38	R/O A small pineal cyst (less than 4mm)
14	2001-08-17	2010-06-12	105	104	103	122,00	4,2	2	1	17,00	134,9	38,0	0,761	1,459	1,565	0,668	1,401	1,538	157,5	-0,73	2,0	1,0	0,19	25,33	3,09	16,95	R choroidal fissure cyst(1cm)
15	2011-02-19	2019-11-22	105	104	96	122,50	4,7	3	1	17,50	137,0	36,5	1,118	1,258	1,048	1,025	1,199	1,021	159,5	-0,32	2,5	1,0	0,20	8,60	1,57	8,14	R/O R choroidal fissure cyst.
16	2011-08-13	2020-05-15	105	103	100	132,00	6,6	4	2	27,00	140,4	39,8	1,769	1,742	1,351	1,581	1,627	1,296	159,5	-0,32	3,0	1,0	3,85	77,83	3,76	16,49	A pineal cyst
17	2011-01-27	2020-01-21	107	107	99	122,50	4,7	3	1	15,50	135,4	31,5	0,575	0,299	0,025	0,575	0,299	0,025	166,5	1,07	2,0	1,0	0,20	15,24	2,84	10,21	R/O Aneurysmal bone cyst or chordoma at sphenoid bone
18	2012-09-17	2018-04-02	66	65	62	95,00	5,5	3	1	29,00	114,8	23,3	0,858	1,383	1,236	0,736	1,301	1,222	157,5	-0,73	2,0	1,0	0,20	7,00	2,76	22,64	
19	2014-01-28	2020-06-16	76	75	74	114,00	6,2	4	2	38,00	127,5	22,5	2,397	0,384	-1,501	2,276	0,312	-1,503	165,0	0,78	2,0	1,0	0,21	19,83	2,14	18,96	
20	2008-02-25	2014-09-19	78	78	68	108,00	4,5	3	1	30,00	124,0	23,5	1,316	0,460	-0,428	1,316	0,460	-0,428	158,5	-0,52	2,0	1,0	0,20	5,34	0,83	16,02	
21	2013-01-05	2019-07-16	78	73	34	86,00	4,3	3	1	8,00	119,4	22,6	0,916	0,564	0,021	0,340	0,197	-0,055	162,5	0,29	2,5	1,0	0,20	5,22	1,21	12,10	
22	2005-03-02	2011-10-15	79	78	77	108,00	3,9	2	1	29,00	118,8	23,0	0,405	0,598	0,481	0,102	0,242	0,462	158,0	-0,63	2,0	1,0	0,38	10,19	1,57	8,84	
23	2005-04-13	2011-12-06	79	78	74	108,00	4,2	3	1	29,00	119,7	24,0	0,211	0,316	0,217	0,295	0,524	0,201	166,5	1,07	1,0	1,0	0,20	9,29	3,76	31,96	
24	2013-08-21	2020-04-02	79	77	76	94,00	5,0	4	1	15,00	121,0	22,5	0,795	0,241	-0,359	0,573	0,092	-0,383	159,0	-0,42	2,0	1,0	0,20	11,42	1,63	25,87	
25	2004-05-07	2011-01-17	80	79	71	122,00	5,3	3	1	42,00	133,0	27,5	3,031	1,355	-0,265	2,906	1,285	-0,278	163,5	0,49	3,5	1,0	0,20	5,68	1,73	10,20	
26	2009-05-22	2016-01-27	80	79	78	101,50	5,6	4	1	21,50	117,2	19,0	-0,245	-1,171	-1,518	-0,352	-1,243	-1,520	159,0	-0,42	2,5	1,0	5,88	8,31	4,68	15,32	
27	2012-03-23	2018-12-06	80	78	77	95,00	4,5	3	1	15,00	121,2	23,8	0,725	0,543	0,160	0,505	0,397	0,128	162,5	0,29	2,5	1,0	1,40	18,60	1,80	35,30	
28	2005-03-14	2011-12-17	81	79	75	108,00	3,9	2	1	27,00	124,7	26,0	1,350	1,025	0,445	1,124	0,884	0,409	158,0	-0,63	1,5	1,0	0,20	7,21	3,51	26,30	
29	2013-09-23	2020-07-03	81	80	78	114,00	5,0	4	1	33,00	121,0	23,7	0,463	0,370	0,120	0,354	0,299	0,104	158,0	-0,63	3,0	1,0	0,20	7,68	0,99	7,31	
30	2005-04-26	2012-03-02	82	80	74	101,50	5,7	2	1	19,50	124,5	25,0	1,195	0,713	0,085	0,972	0,573	0,055	158,0	-0,63	1,5	1,0	0,20	7,74	1,88	12,16	
31	2013-07-05	2020-05-07	82	81	74	108,00	5,5	4	1	26,00	130,7	34,4	2,333	2,382	1,982	2,215	2,318	1,953	167,0	1,17	2,5	1,0	0,20	8,58	0,61	8,54	
32	2004-01-21	2011-01-13	83	83	82	108,00	8,6	4	2	25,00	122,0	30,0	0,349	1,565	1,930	0,349	1,565	1,930	159,0	-0,42	2,0	1,0	0,20	8,95	3,23	19,58	
33	2005-04-22	2012-04-17	83	83	79	108,00	4,7	3	1	25,00	121,0	26,0	0,140	0,747	0,907	0,140	0,747	0,907	160,0	-0,21	2,5	1,0	0,20	6,45	1,46	17,04	
34	2009-02-28	2016-02-25	83	82	80	108,00	5,0	2	1	25,00	121,8	23,5	0,414	0,173	-0,119	0,307	0,105	-0,132	160,0	-0,21	2,0	1,0	0,20	7,24	1,51	17,62	
35	2010-01-11	2016-12-26	83	82	80	108,00	7,5	4	2	25,00	127,7	27,3	1,620	1,105	0,403	1,512	1,038	0,385	159,5	-0,32	2,5	1,0	0,20	7,10	2,30	11,60	
36	2010-03-22	2017-04-06	84	84	71	86,00	4,1	3	1	2,00	121,0	27,3	0,037	0,967	1,286	0,037	0,967	1,286	161,5	0,09	2,5	1,0	0,20	7,14	1,09	12,15	
37	2004-07-10	2011-09-09	85	84	78	108,00	7,0	4	2	23,00	127,0	27,0	1,260	0,902	0,359	1,153	0,832	0,335	161,0	-0,01	3,0	1,0	0,20	7,13	2,35	17,76	
38	2007-05-31	2014-07-28	85	83	83	108,00	6,2	4	2	23,00	127,7	25,5	1,512	0,628	-0,257	1,291	0,486	-0,299	159,5	-0,32	2,0	1,0	0,20	6,23	0,56	5,86	
39	2004-08-01	2011-10-13	86	84	82	125,00	4,5	3	1	39,00	122,6	26,5	0,369	0,791	0,817	0,165	0,651	0,763	158,5	-0,52	2,0	1,0	0,20	8,38	1,50	10,72	
40	2007-06-13	2014-08-29	86	84	83	95,00	6,8	4	2	9,00	121,2	26,0	0,079	0,676	0,851	-0,124	0,536	0,796	153,5	-1,58	2,0	1,0	0,30	7,75	1,19	13,99	
41	2011-01-05	2018-03-23	86	85	84	108,00	3,4	2	1	22,00	124,0	31,0	0,348	0,765	0,798	0,449	1,535	0,771	159,5	-0,32	3,0	1,0	0,20	5,63	1,57	18,75	
42	2011-01-24	2018-03-29	86	85	83	115,25	3,3	2	1	29,25	123,0	26,7	0,552	1,602	1,869	0,246	0,697	1,838	162,5	0,29	2,5	1,0	0,27	7,53	1,65	10,85	
43	2012-01-23	2019-03-29	86	85	82	108,00	7,2	5	2	22,00	126,3	25,4	1,014	0,461	-0,127	0,910	0,392	-0,148	158,5	-0,52	3,5	1,0	0,20	5,01	1,01	11,50	
44	2012-04-27	2019-06-29	86	85	83	108,00	4,8	3	1	22,00	118,2	20,7	-0,655	-0,930	-0,831	-0,755		-0,847	157,5	-0,73	2,5	1,0	0,20	5,48	1,42	10,17	
45	2013-03-27	2020-06-15	86	85	84	102,00	5,0	3	1	16,00	131,2	25,2	1,970	0,412	-0,952	1,860	0,342	-0,967	164,5	0,69	2,5	1,0	0,20	13,88	1,70	8,98	
46	2013-03-26	2020-06-18	86	86	84	108,00	5,5	4	1	22,00	128,1	31,0	1,263	1,535	1,333	1,263	1,535	1,333	167,0	1,17	2,0	1,0	0,20	6,00	3,12	20,04	
47	2006-06-28	2013-10-26	87	87	86	108,00	4,6	2	1	21,00	123,2	23,5	0,186	-0,177	-0,434	0,186	-0,177	-0,434	157,5	-0,73	2,5	1,0	0,20	6,14	2,34	17,15	
48	2013-01-30	2020-05-27	87	87	85	120,00	5,3	3	1	33,00	128,7	28,4	1,275	0,990	0,496	1,275	0,990	0,496	164,0	0,59	2,0	1,0	0,20	22,63	0,79	5,42	
49	2008-12-12	2016-04-30	88	87	85	108,00	9,0	4	2	20,00	133,0	28,5	0,944	2,189	2,497	1,980	0,943	2,464	163,0	0,39	3,0	1,0	0,20	5,05	1,83	10,96	
50	2010-01-05	2017-05-23	88	86	80	108,00	5,2	3	1	20,00	130,2	31,6	2,422	0,828	-0,620	1,457	1,507	-0,655	163,0	0,39	3,0	1,0	0,27	5,45	2,60	17,71	
51	2010-11-15	2018-03-22	88	87	75	108,00	4,7	2	1	20,00	127,0	35,8	1,562	1,571	1,196	0,842	2,127	1,168	160,5	-0,11	3,0	1,0	0,76	20,10	1,57	6,46	
52	2012-05-31	2019-10-12	88	86	86	122,50	4,6	3	1	34,50	122,2	25,9	0,083	0,512	0,622	-0,118	0,378	0,570	155,0	-1,26	2,5	1,0	0,20	5,70	2,70	14,60	
53	2003-04-03	2010-09-13	89	88	84	108,00	5,2	3	1	19,00	133,1	26,5	1,998	0,517	-0,784	1,892	0,452	-0,799	160,0	-0,21	3,0	1,0	0,32	24,04	2,66	8,29	
54	2004-02-11	2011-07-26	89	88	87	122,00	5,5	3	1	33,00	129,7	26,0	1,362	0,401	-0,469	1,259	0,336	-0,487	162,0	0,19	2,5	1,0	0,20	6,01	0,61	10,10	
55	2010-06-21	2017-11-27	89	86	83	108,00	5,4	4	1	19,00	120,5	21,4	2,030	0,629	-0,639	-0,568	-0,958	-0,690	159,0	-0,42	2,0	1,0	0,50	12,84	1,25	7,63	
56	2012-02-07	2019-08-02	89	88	88	127,25	5,4	3	1	38,25	132,1	26,4	-0,469	-0,892	-0,930	1,708	0,429	-0,944	160,5	-0,11	2,5	1,0	0,20	15,64	2,68	19,71	
57	2013-01-08	2020-07-03	89	89	87	120,00	3,6	3	1	31,00	128,8	26,9	1,088	0,542	-0,045	1,088	0,542	-0,045	164,5	0,69	2,0	1,0	0,20	7,29	1,59	16,83	
58	2013-02-06	2020-07-18	89	87	85	108,00	5,6	4	1	19,00	126,2	29,5	0,787	1,203	1,145	0,585	1,073	1,088	161,0	-0,01	2,0	1,0	0,20	18,39	1,05	9,63	
59	2002-11-09	2010-05-13	90	88	86	108,00	4,0	2	1	18,00	126,0	23,0	0,646	-0,389	-1,101	0,447	-0,525	-1,127	163,0	0,39	2,5	1,0	0,22	6,34	2,23	12,80	
60	2004-07-22	2012-02-21	90	88	85	132,00	4,2	3	1	42,00	133,0	28,0	1,152	0,281	-0,491	1,770	0,708	-0,528	163,0	0,39	1,5	1,0	0,20	5,99	1,47	12,99	
61	2006-08-30	2014-03-07	90	89	87	108,00	5,6	4	1	18,00	128,6	25,5	2,218	1,342	0,390	0,950	0,146	0,367	157,5	-0,73	2,0	1,0	0,20	17,19	0,60	9,24	
62	2011-05-03	2018-11-09	90	89	85	108,00	7,1	5	2	18,00	124,0	24,1	-0,013	0,166	0,201	0,050	-0,214	0,178	157,5	-0,73	2,5	1,0	0,52	6,59	1,76	9,52	
63	2011-10-27	2019-05-16	90	89	89	122,50	4,3	3	1	32,50	123,2	25,3	0,148	-0,145	-0,356	-0,111	0,097	-0,375	155,0	-1,26	2,5	1,0	0,20	8,56	1,60	13,02	
64	2012-06-26	2020-01-09	90	89	81	122,50	4,3	3	1	32,50	134,5	35,0	2,146	1,959	1,432	2,042	1,895	1,404	163,0	0,39	3,0	1,0	0,20	8,11	1,43	7,46	
65	2012-09-06	2020-03-09	90	89	87	132,00	6,0	4	2	42,00	135,4	30,6	2,308	1,273	0,213	2,202	1,206	0,190	159,0	-0,42	3,0	1,0	0,20	9,05	3,13	8,39	
66	2006-11-22	2014-07-03	91	90	89	122,50	4,7	3	1	31,50	127,7	31,0	0,777	1,275	1,267	0,677	1,210	1,239	161,0	-0,01	2,0	1,0	0,20	7,83	0,66	18,60	
67	2007-04-10	2014-11-19	91	91	82	132,00	4,9	3	1	41,00	131,7	35,0	1,427	1,833	1,692	1,427	1,833	1,692	165,0	0,78	2,5	1,0	0,20	5,28	2,54	13,88	
68	2009-09-18	2017-04-26	91	90	89	108,00	4,0	3	1	17,00	129,1	26,1	1,045	0,290	-0,384	0,943	0,221	-0,402	160,5	-0,11	2,0	1,0	0,25	10,07	2,39	16,29	
69	2011-02-10	2018-10-04	91	90	88	108,00	5,1	3	1	17,00	125,1	24,4	1,843	0,850	-0,115	0,171	-0,203	-0,135	151,5	-2,02	2,0	1,0	0,34	18,84	1,03	13,06	
70	2011-04-29	2018-12-14	91	90	87	122,50	4,9	3	1	31,50	133,4	28,7	0,269	-0,134	-0,424	1,737	0,783	-0,442	158,5	-0,52	3,0	1,0	1,02	13,24	1,44	10,74	
71	2012-05-06	2019-12-17	91	90	88	122,50	4,6	3	1	31,50	126,2	28,3	0,486	0,770	0,723	0,387	0,703	0,698	156,0	-1,05	2,0	1,0	0,20	20,29	2,06	14,21	
72	2012-11-07	2020-06-09	91	89	86	132,00	5,4	4	1	41,00	124,0	26,7	0,148	0,497	0,555	-0,047	0,360	0,506	159,0	-0,42	3,0	1,0	0,27	18,06	0,77	6,18	
73	2002-12-03	2010-08-28	92	91	91	122,00	3,9	2	1	30,00	130,4	30,0	1,773	1,032	0,228	1,088	0,966	0,205	155,0	-1,26	2,0	1,0	0,20	5,18	2,95	16,98	
74	2005-03-16	2012-12-05	92	92	88	108,00	6,0	4	2	16,00	133,6	30,0	1,088	0,966	0,614	1,672	0,966	0,614	157,0	-0,84	2,5	1,0	0,20	5,56	0,91	16,69	
75	2005-12-28	2013-09-05	92	90	82	132,00	4,2	3	1	40,00	133,0	32,0	0,348	0,218	0,024	1,564	1,315	-0,018	165,5	0,88	2,5	1,0	0,20	11,94	0,50	7,68	
76	2006-07-05	2014-03-10	92	90	89	132,00	5,6	3	1	40,00	129,0	26,0	1,770	1,443	0,871	0,826	0,131	0,818	158,5	-0,52	2,0	1,0	0,20	9,76	1,88	7,40	
77	2007-09-03	2015-05-04	92	88	88	115,25	3,5	3	1	23,25	135,4	29,0	1,229	0,401	-0,367	1,992	0,776	-0,441	162,5	0,29	1,5	1,0	0,20	8,99	2,59	16,69	
78	2009-04-16	2017-01-13	92	92	91	115,25	4,0	3	1	23,25	131,3	33,8	1,992	0,776	-0,329	1,255	1,597	-0,329	160,5	-0,11	3,0	1,0	0,25	16,64	3,90	11,82	
79	2009-05-26	2017-02-20	92	91	89	108,00	5,0	2	1	16,00	128,8	38,2	2,076	2,259	1,956	0,789	2,188	1,925	164,0	0,59	2,0	1,0	0,33	5,56	1,86	10,87	
80	2009-06-22	2017-03-17	92	92	85	132,00	4,0	3	1	40,00	135,3	38,3	1,255	1,597	1,447	1,974	2,200	1,447	160,5	-0,11	2,5	1,0	0,20	21,71	1,33	11,32	
81	2010-11-26	2018-07-26	92	90	87	108,00	6,0	4	2	16,00	134,6	29,7	0,988	2,306	2,650	1,850	0,910	2,586	162,5	0,29	3,0	1,0	0,20	6,64	1,67	10,03	
82	2012-03-17	2019-11-22	92	90	90	108,00	5,8	4	1	16,00	127,2	23,5	0,681	-0,380	-1,102	0,484	-0,516	-1,127	163,0	0,39	2,0	1,0	0,20	6,83	1,48	16,20	
83	2012-09-05	2020-05-06	92	91	91	108,00	4,3	3	1	16,00	129,8	32,9	1,075	1,523	1,446	0,977	1,459	1,417	162,5	0,29	2,5	1,0	0,20	15,01	0,70	11,50	
84	2012-10-28	2020-07-02	92	88	87	120,00	3,9	3	1	28,00	130,8	26,9	1,570	0,607	-0,308	1,162	0,338	-0,383	162,0	0,19	2,5	1,0	0,30	6,70	3,06	17,40	
85	2005-12-02	2013-09-10	93	92	92	108,00	4,9	3	1	15,00	123,3	28,0	-0,144	-0,660	-0,845	-0,380	0,510	-0,860	157,0	-0,84	2,0	1,0	0,37	14,78	2,59	12,78	
86	2006-05-25	2014-03-19	93	93	93	108,00	4,3	3	1	15,00	125,7	27,5	-0,380	0,510	0,936	0,096	0,404	0,936	157,0	-0,84	2,0	1,0	0,20	22,15	1,84	18,30	
87	2007-11-13	2015-08-25	93	91	89	101,50	4,3	3	1	8,50	121,3	22,0	0,289	0,536	0,525	-0,787	-1,032	0,477	161,5	0,09	2,5	1,0	0,20	8,88	2,51	19,51	
88	2007-11-20	2015-09-02	93	93	82	101,50	4,8	3	1	8,50	124,0	23,0	-0,787	-1,032	-0,864	-0,239	-0,727	-0,864	156,5	-0,94	2,5	1,0	0,42	18,76	0,76	11,84	
89	2009-06-04	2017-03-30	93	93	91	115,25	4,0	2	1	22,25	125,5	28,0	1,751	0,957	0,141	0,057	0,510	0,141	160,0	-0,21	2,0	1,0	0,20	5,80	1,57	8,90	
90	2009-10-30	2017-07-31	93	91	86	127,25	5,0	3	1	34,25	134,6	30,3	0,250	0,641	0,701	1,751	0,957	0,652	162,5	0,29	2,0	1,0	0,20	5,31	0,88	6,56	
91	2011-04-25	2019-02-22	93	92	92	108,00	7,1	5	2	15,00	127,1	28,6	0,464	0,697	0,642	0,368	0,632	0,618	157,0	-0,84	2,0	1,0	0,20	5,68	2,05	21,01	
92	2012-01-12	2019-11-06	93	91	79	127,25	5,3	3	1	34,25	127,3	29,2	0,600	0,881	0,812	0,406	0,751	0,761	161,5	0,09	3,0	1,0	0,20	10,61	1,78	7,81	
93	2003-04-07	2011-02-21	94	91	89	107,00	4,7	3	1	13,00	135,7	33,5	0,811	1,032	0,892	1,844	1,428	0,815	160,5	-0,11	2,0	1,0	0,20	5,14	1,39	14,46	
94	2003-05-09	2011-03-11	94	93	73	102,00	4,0	3	1	8,00	141,1	40,0	2,859	2,340	1,602	2,756	2,283	1,573	163,0	0,39	3,5	1,0	0,20	9,98	4,12	17,77	
95	2003-11-21	2011-10-14	94	93	91	108,00	4,7	3	1	14,00	139,8	37,0	1,944	1,490	0,838	2,541	1,920	0,813	161,5	0,09	2,5	1,0	0,35	5,41	2,05	17,83	
96	2003-12-14	2011-10-27	94	93	92	132,00	5,8	3	1	38,00	130,9	28,0	2,644	1,980	1,152	0,986	0,445	1,125	157,5	-0,73	2,5	1,0	0,20	8,18	2,19	21,88	
97	2004-06-30	2012-05-16	94	94	94	108,00	4,7	3	1	14,00	128,4	30,0	0,986	0,445	-0,080	0,520	0,839	-0,080	159,0	-0,42	2,0	1,0	0,20	13,60	5,31	20,54	
98	2005-07-25	2013-06-22	94	93	92	108,00	4,2	2	1	14,00	126,7	29,5	0,291	0,808	0,919	0,195	0,744	0,893	154,5	-1,37	2,0	1,0	0,20	16,76	0,69	7,97	
99	2007-11-18	2015-10-13	94	94	91	108,00	4,5	3	1	14,00	125,0	28,5	1,078	0,548	0,007	-0,136	0,548	0,007	161,5	0,09	2,5	1,0	0,20	10,03	2,23	11,74	
100	2008-07-06	2016-05-10	94	92	91	127,25	5,6	4	1	33,25	131,4	28,5	0,055	0,677	0,884	1,078	0,548	0,834	163,0	0,39	3,0	1,0	0,20	13,24	0,80	10,52	
101	2010-04-17	2018-03-08	94	94	91	108,00	5,5	4	1	14,00	129,6	26,8	1,115	0,070	-0,735	0,746	0,185	-0,735	156,5	-0,94	3,0	1,0	0,64	22,57	1,18	11,76	
102	2011-03-25	2019-02-06	94	94	93	95,00	4,7	3	1	1,00	131,6	26,3	0,746	0,185	-0,289	1,115	0,070	-0,289	162,0	0,19	2,5	1,0	0,20	5,15	2,69	15,41	
103	2012-03-12	2020-02-04	94	93	92	122,50	5,1	3	1	28,50	124,7	23,4	-0,100	-0,611	-0,804	-0,196	-0,675	-0,819	159,8	-0,26	2,0	1,0	0,20	6,38	1,12	17,17	
104	2012-04-04	2020-02-07	94	92	90	122,50	5,2	4	1	28,50	123,1	31,7	-0,324	1,265	1,924	-0,515	1,139	1,866	155,0	-1,26	3,0	1,0	0,20	19,53	1,63	7,98	
105	2002-04-12	2010-04-09	95	95	87	108,00	4,4	1	1	13,00	127,8	28,0	0,482	0,585	0,480	0,311	0,382	0,480	155,0	-1,26	3,0	1,0	6,60	15,76	3,83	7,54	
106	2002-12-23	2010-12-14	95	94	93	108,00	5,0	3	1	13,00	128,7	29,0	1,757	1,578	1,102	0,482	0,585	1,076	157,5	-0,73	2,5	1,0	0,20	15,38	2,22	16,65	
107	2003-01-25	2011-01-21	95	95	89	122,50	5,0	3	1	27,50	135,2	34,5	2,174	1,590	0,846	1,660	1,518	0,846	156,5	-0,94	2,5	1,0	0,82	69,70	3,08	17,80	
108	2003-03-04	2011-02-18	95	89	83	108,00	6,5	4	2	13,00	138,2	35,0	1,145	1,509	1,372	2,174	1,590	1,207	163,0	0,39	3,0	1,0	0,20	6,69	2,86	14,85	
109	2003-06-03	2011-05-09	95	93	86	108,00	3,9	2	1	13,00	133,8	35,0	0,502	0,510	0,351	1,413	1,590	0,306	158,0	-0,63	2,0	1,0	0,72	19,23	2,19	17,55	
110	2003-07-29	2011-07-08	95	95	81	108,00	3,1	2	1	13,00	129,7	29,0	1,413	1,590	1,344	0,669	0,585	1,344	157,5	-0,73	2,5	2,0	0,48	18,08	3,68	9,59	
111	2006-01-28	2014-01-14	95	94	94	108,00	5,0	3	1	13,00	124,9	27,0	0,764	0,648	0,375	-0,251	0,166	0,352	158,5	-0,52	2,0	1,0	0,20	7,80	1,77	16,30	
112	2006-02-21	2014-01-22	95	90	90	132,00	5,7	4	1	37,00	129,1	32,0	0,230	0,495	0,503	0,557	1,128	0,385	158,0	-0,63	2,5	1,0	0,20	5,72	2,42	20,77	
113	2006-10-31	2014-10-02	95	94	91	108,00	3,6	2	1	13,00	130,7	28,0	0,950	0,445	-0,053	0,854	0,382	-0,073	160,0	-0,21	2,0	1,0	0,20	11,78	2,80	15,61	
114	2010-06-23	2018-05-26	95	93	89	122,50	5,0	5	1	27,50	136,6	36,7	2,100	1,941	1,444	1,902	1,823	1,389	156,0	-1,05	2,5	1,0	0,28	5,80	2,65	11,91	
115	2011-04-19	2019-04-02	95	91	89	132,00	5,0	2	1	37,00	135,8	30,5	2,164	1,122	0,087	1,764	0,868	0,004	163,5	0,49	2,5	1,0	0,20	5,07	2,00	15,26	
116	2011-12-11	2019-11-19	95	92	88	122,50	3,1	2	1	27,50	133,6	27,1	1,672	0,382	-0,705	1,378	0,188	-0,752	165,0	0,78	3,0	1,0	0,70	17,20	2,56	12,14	
117	2012-01-28	2020-01-18	95	94	92	108,00	5,7	4	1	13,00	121,5	23,7	-0,841	-0,591	-0,234	-0,936	-0,655	-0,253	156,0	-1,05	2,0	1,0	1,57	45,89	2,59	15,86	
118	2012-04-01	2020-03-20	95	93	85	120,00	4,6	2	1	25,00	127,8	27,7	0,502	0,446	0,260	0,311	0,319	0,216	156,5	-0,94	3,0	1,0	0,20	6,86	0,84	10,61	
119	2012-04-27	2020-04-22	95	95	93	126,00	5,1	3	1	31,00	129,9	26,0	0,706	-0,064	-0,618	0,706	-0,064	-0,618	157,5	-0,73	2,0	1,0	0,20	31,63	0,78	19,50	
120	2012-05-24	2020-05-03	95	94	92	108,00	6,0	4	2	13,00	139,0	37,0	2,408	1,920	1,213	2,308	1,862	1,187	167,5	1,27	2,0	1,0	0,20	17,11	2,74	15,15	
121	2004-12-26	2013-01-18	96	93	91	108,00	5,1	3	1	12,00	123,9	23,5	-0,542	0,612	1,177	-0,543	-0,778	1,094	153,5	-1,58	2,0	1,0	0,20	6,94	2,96	15,99	
122	2005-02-27	2013-03-16	96	95	91	127,25	5,8	4	1	31,25	128,0	24,5	0,349	-0,438	-0,892	0,254	-0,506	-0,912	160,0	-0,21	2,0	1,0	0,20	7,72	1,06	12,97	
123	2010-04-05	2018-04-06	96	95	95	108,00	4,7	2	1	12,00	124,3	25,8	-0,370	-0,112	0,085	-0,463	-0,179	0,059	158,0	-0,63	2,0	1,0	0,52	12,03	1,21	11,22	
124	2010-07-14	2018-08-01	96	96	92	108,00	4,9	3	1	12,00	130,1	26,2	0,646	-0,084	-0,599	0,646	-0,084	-0,599	159,0	-0,42	3,0	1,0	0,20	5,25	0,79	10,79	
125	2011-04-04	2019-04-20	96	94	94	108,00	4,5	3	1	12,00	128,0	30,5	0,444	0,930	0,994	0,254	0,803	0,940	169,5	1,64	2,5	1,0	0,20	9,23	3,25	10,04	
126	2011-07-09	2019-07-11	96	95	95	115,25	3,6	3	1	19,25	122,3	21,6	-0,772	-1,287	-1,219	-0,865	-1,353	-1,236	158,5	-0,52	2,5	1,0	0,20	8,66	2,28	14,06	
127	2011-09-22	2019-10-11	96	95	91	132,00	4,1	3	1	36,00	136,2	37,1	1,833	1,875	1,512	1,731	1,815	1,482	163,5	0,49	2,5	1,0	0,64	21,03	2,77	11,13	
128	2012-03-13	2020-04-10	96	96	90	108,00	4,8	3	1	12,00	125,3	27,4	-0,266	0,187	0,426	-0,266	0,187	0,426	159,0	-0,42	3,0	1,0	0,20	15,60	1,40	10,29	
129	2012-05-29	2020-06-08	96	94	93	108,00	3,2	1	1	12,00	129,0	25,2	0,633	-0,195	-0,761	0,442	-0,326	-0,797	162,0	0,19	2,5	1,0	0,44	7,75	1,50	16,84	
130	2002-04-27	2010-06-14	97	97	93	108,00	4,7	2	1	11,00	128,4	25,0	0,046	0,144	0,155	0,235	-0,442	0,155	159,5	-0,32	2,5	1,0	0,20	17,31	0,79	15,71	
131	2004-07-06	2012-08-07	97	94	94	101,50	5,7	4	1	4,50	135,0	25,0	1,722	-0,245	-1,709	1,429	-0,442	-1,743	163,5	0,49	1,0	1,0	0,20	17,04	0,51	6,47	
132	2005-06-13	2013-08-01	97	88	87	132,00	4,7	3	1	35,00	128,0	24,5	1,113	0,157	-0,651	0,160	-0,571	-0,805	157,5	-0,73	3,5	1,0	0,20	29,52	2,08	16,94	
133	2006-09-10	2014-11-03	97	97	95	132,00	6,0	3	2	35,00	131,0	25,0	0,084	-0,651	-0,987	0,717	-0,442	-0,987	158,5	-0,52	2,0	1,0	0,20	5,03	1,12	8,59	
134	2006-09-21	2014-11-10	97	97	90	108,00	5,2	4	1	11,00	127,4	27,5	0,160	-0,571	-0,932	0,046	0,144	-0,932	157,5	-0,73	3,0	1,0	0,20	5,13	0,81	7,93	
135	2010-12-18	2019-02-03	97	94	84	127,25	4,9	3	1	30,25	127,6	24,2	1,005	-0,245	-1,123	0,084	-0,651	-1,172	161,0	-0,01	3,0	1,0	0,24	7,80	1,79	7,00	
136	2011-02-05	2019-03-29	97	96	95	108,00	6,2	4	2	11,00	138,0	28,0	2,270	2,419	2,086	1,940	0,250	2,058	159,0	-0,42	3,0	1,0	0,21	11,73	1,70	9,81	
137	2011-03-04	2019-05-02	97	96	87	132,00	5,1	3	1	35,00	139,4	42,3	2,037	0,315	-1,068	2,172	2,362	-1,087	162,0	0,19	3,0	1,0	0,20	8,22	1,42	8,02	
138	2012-03-01	2020-04-07	97	93	89	122,70	4,2	2	1	25,70	129,8	29,9	0,879	0,884	0,638	0,497	0,627	0,534	162,5	0,29	2,5	1,0	1,47	18,68	2,88	16,30	
139	2012-04-02	2020-05-27	97	96	90	114,00	6,2	4	2	17,00	132,5	30,4	1,083	0,784	0,362	0,988	0,720	0,335	167,5	1,27	3,0	1,0	0,20	6,71	0,97	11,38	
140	2002-06-08	2010-08-12	98	93	81	108,00	4,6	3	1	10,00	129,2	22,5	0,954	0,902	0,615	0,292	-1,200	0,484	164,0	0,59	2,5	1,0	0,34	10,13	1,42	4,92	
141	2004-12-01	2013-02-13	98	96	94	127,25	4,6	3	1	29,25	130,2	30,0	0,479	-1,070	-1,902	0,477	0,582	-1,924	158,5	-0,52	1,5	1,0	0,53	17,69	3,08	17,56	
142	2007-04-16	2015-07-08	98	96	88	115,25	4,7	3	1	17,25	132,6	30,5	1,101	0,803	0,376	0,912	0,675	0,323	153,5	-1,58	3,0	1,0	0,20	5,14	2,17	16,67	
143	2008-05-06	2016-07-20	98	97	88	108,00	7,0	5	2	10,00	131,2	28,7	1,271	0,828	0,298	0,660	0,330	0,272	160,5	-0,11	3,0	1,0	0,43	34,60	3,12	16,10	
144	2010-11-22	2019-02-21	98	98	93	138,00	7,0	5	2	40,00	126,8	27,1	1,001	0,564	0,098	-0,163	-0,009	0,098	161,5	0,09	3,0	1,0	0,57	17,86	3,32	9,76	
145	2011-02-26	2019-05-13	98	96	95	122,50	6,1	4	2	24,50	133,1	29,9	0,025	0,122	0,138	1,001	0,564	0,087	160,0	-0,21	2,0	1,0	0,20	10,41	2,21	10,30	
146	2011-06-05	2019-08-27	98	98	98	127,25	3,2	2	1	24,25	123,2	29,5	-0,871	0,487	1,211	-0,871	0,487	1,211	159,5	-0,32	2,5	1,0	0,30	5,90	1,60	20,20	
147	2012-03-22	2020-06-19	98	97	94	120,00	3,6	2	1	22,00	125,2	28,3	-0,380	0,312	0,671	-0,473	0,248	0,643	157,5	-0,73	2,5	1,0	0,20	6,56	1,03	8,19	
148	2012-04-23	2020-07-14	98	97	94	108,00	4,8	2	1	10,00	127,5	26,9	0,065	0,011	-0,043	-0,029	-0,053	-0,067	161,5	0,09	2,0	1,0	0,57	6,37	0,70	10,56	
149	2002-11-11	2011-03-02	99	99	97	122,50	5,3	4	1	23,50	138,5	35,0	2,079	0,122	-1,386	1,833	1,345	-1,386	164,0	0,59	3,0	1,0	1,20	18,69	2,26	4,84	
150	2008-03-25	2016-06-27	99	98	90	132,00	5,5	3	1	33,00	135,2	36,8	1,928	1,405	0,727	1,275	1,596	0,699	157,0	-0,84	3,5	1,0	0,38	34,43	3,09	20,43	
151	2009-10-19	2018-02-01	99	98	81	132,00	5,5	4	1	33,00	140,0	28,0	1,334	0,903	0,370	2,079	0,122	0,344	158,0	-0,63	3,0	1,0	0,30	28,00	1,00	13,80	
152	2011-05-01	2019-08-25	99	99	93	122,50	3,8	3	1	23,50	135,0	31,8	1,275	1,596	1,444	1,240	0,842	1,444	160,8	-0,05	2,5	1,0	0,55	15,85	1,91	7,10	
153	2011-06-17	2019-10-02	99	98	93	122,50	4,3	2	1	23,50	131,2	27,9	0,660	0,165	-0,243	0,567	0,101	-0,266	157,5	-0,73	3,0	1,0	0,20	6,99	2,24	12,83	
154	2012-03-05	2020-06-23	99	97	95	120,00	5,2	4	1	21,00	130,5	29,9	0,626	0,627	0,447	0,439	0,501	0,394	160,3	-0,15	2,5	1,0	2,78	20,23	0,89	9,93	
155	2003-04-02	2011-08-22	100	100	98	132,00	5,6	3	1	32,00	132,9	29,0	0,655	0,265	-0,093	0,780	0,265	-0,093	161,0	-0,01	2,0	1,0	0,26	26,25	1,88	13,93	
156	2003-06-25	2011-10-27	100	97	93	122,50	4,6	3	1	22,50	140,8	37,5	0,626	0,828	0,736	2,114	1,630	0,654	162,0	0,19	2,5	1,0	0,20	5,67	1,29	22,55	
157	2004-02-15	2012-06-20	100	97	93	108,00	4,7	3	1	8,00	130,5	31,0	2,401	1,806	1,035	0,347	0,641	0,950	161,0	-0,01	2,5	1,0	0,37	15,39	2,38	15,00	
158	2005-06-23	2013-10-23	100	99	93	132,00	5,0	3	1	32,00	138,6	40,0	0,872	0,327	-0,157	1,756	1,939	-0,181	147,0	-3,05	3,0	1,0	0,20	17,93	2,93	9,25	
159	2008-11-07	2017-04-06	100	100	96	132,00	6,4	4	2	32,00	141,0	39,0	-0,897	4,701	6,745	2,146	1,819	6,745	158,0	-0,63	2,5	1,0	1,49	69,24	4,41	13,05	
160	2009-06-16	2017-10-17	100	98	89	127,25	5,8	3	1	27,25	130,3	26,7	0,822	0,601	0,277	0,310	-0,224	0,225	160,5	-0,11	2,5	1,0	0,20	5,75	1,04	8,96	
161	2009-07-16	2017-11-17	100	99	97	122,50	5,5	4	1	22,50	124,0	84,0	2,241	1,876	1,252	-0,897	4,701	1,224	156,5	-0,94	2,0	1,0	0,61	16,79	2,33	15,32	
162	2010-12-14	2019-04-29	100	99	96	132,00	4,7	2	1	32,00	131,8	38,2	0,402	-0,162	-0,525	0,583	1,720	-0,547	157,0	-0,84	3,0	1,0	0,20	7,45	0,64	7,20	
163	2011-02-16	2019-07-12	100	99	93	122,50	5,0	3	1	22,50	132,1	30,1	-1,626	0,016	1,050	0,637	0,477	1,023	160,0	-0,21	2,5	1,0	0,49	5,35	3,18	12,27	
164	2011-04-15	2019-08-16	100	99	92	114,00	5,0	3	1	14,00	120,0	27,5	0,675	1,777	2,070	-1,720	-0,047	2,042	151,5	-2,02	2,5	1,0	0,20	12,72	1,73	6,82	
165	2011-09-13	2020-01-18	100	99	90	122,50	4,1	3	1	22,50	134,0	38,3	1,066	1,790	1,858	0,974	1,732	1,829	158,5	-0,52	3,0	1,0	0,20	10,81	3,60	13,42	
166	2011-11-08	2020-03-12	100	99	98	120,00	5,2	3	1	20,00	133,9	34,0	1,049	1,196	0,997	0,956	1,136	0,969	160,0	-0,21	2,0	1,0	0,20	10,37	3,19	12,68	
167	2012-03-01	2020-07-07	100	97	95	111,00	5,3	3	1	11,00	128,4	32,2	0,235	1,033	1,277	-0,043	0,848	1,191	162,0	0,19	2,5	1,0	0,55	24,98	2,84	9,20	
168	2012-02-21	2020-07-13	100	98	90	120,00	6,4	4	2	20,00	134,5	30,7	1,247	0,711	0,143	1,061	0,587	0,093	163,0	0,39	3,0	1,0	0,20	5,60	0,63	6,40	
169	2005-02-17	2013-08-06	101	99	85	108,00	5,3	2	1	7,00	133,0	26,0	1,361	0,959	0,436	0,706	-0,449	0,383	161,0	-0,01	2,5	1,0	0,20	10,64	0,85	8,97	
170	2006-05-31	2014-11-14	101	100	93	127,25	4,9	3	1	26,25	135,7	32,5	0,797	-0,387	-1,143	1,176	0,838	-1,159	161,0	-0,01	3,0	1,0	0,20	9,90	2,16	7,73	
171	2011-07-28	2020-01-06	101	100	100	122,50	4,5	3	1	21,50	137,7	37,1	1,606	1,577	1,205	1,513	1,520	1,176	168,0	1,36	2,0	1,0	0,20	6,65	2,39	9,67	
172	2011-09-17	2020-02-26	101	98	90	108,00	3,5	2	1	7,00	127,4	27,8	-0,048	0,143	0,219	-0,325	-0,044	0,143	160,5	-0,11	3,0	1,0	0,20	8,29	1,42	12,07	
173	2011-10-01	2020-03-20	101	100	97	120,00	4,9	3	1	19,00	131,4	29,9	0,511	0,439	0,257	0,419	0,378	0,232	162,0	0,19	2,5	1,0	0,20	6,45	3,40	10,62	
174	2011-10-16	2020-03-26	101	100	96	132,00	8,0	5	2	31,00	137,8	38,4	1,623	1,745	1,448	1,530	1,688	1,418	164,5	0,69	2,5	1,0	0,20	53,05	8,13	23,45	
175	2012-01-16	2020-06-20	101	99	95	120,00	4,9	3	1	19,00	135,7	29,2	1,361	0,366	-0,454	1,176	0,243	-0,497	163,5	0,49	2,5	1,0	0,20	19,72	2,83	15,47	
176	2002-01-31	2010-08-16	102	99	93	122,50	6,0	2	2	20,50	128,1	27,0	1,066	0,520	-0,011	-0,285	-0,283	-0,084	157,5	-0,73	2,5	1,0	0,10	9,42	0,96	13,28	
177	2002-02-06	2010-09-03	102	101	97	115,25	4,3	3	1	13,25	134,6	30,0	0,986	0,397	-0,134	0,893	0,333	-0,157	163,0	0,39	2,0	1,0	0,20	5,38	2,78	13,80	
178	2002-03-25	2010-10-18	102	101	98	122,50	4,1	2	1	20,50	136,0	35,0	-0,192	-0,218	-0,186	1,134	1,165	-0,210	162,0	0,19	2,0	1,0	0,20	11,70	2,12	17,79	
179	2002-06-13	2011-01-11	102	102	96	122,50	4,0	2	1	20,50	134,0	30,0	1,134	1,165	0,897	0,789	0,333	0,897	157,0	-0,84	3,0	1,0	0,20	27,70	2,18	14,04	
180	2006-02-18	2014-08-21	102	101	90	127,25	4,9	2	1	25,25	131,3	27,0	2,052	0,754	-0,371	0,308	-0,283	-0,393	157,5	-0,73	2,0	1,0	0,35	31,08	1,86	17,89	
181	2009-02-15	2017-08-31	102	102	96	132,00	5,0	3	1	30,00	135,7	27,8	2,428	1,812	1,023	1,083	-0,109	1,023	165,0	0,78	3,0	1,0	0,35	20,02	1,77	12,36	
182	2009-08-26	2018-03-08	102	101	95	132,00	6,2	4	2	30,00	132,4	29,3	0,401	-0,218	-0,603	0,506	0,198	-0,624	161,5	0,09	3,0	1,0	0,54	47,55	1,59	15,48	
183	2010-09-04	2019-03-29	102	101	96	127,25	6,5	5	2	25,25	133,1	36,0	0,599	0,263	-0,056	0,630	1,308	-0,080	154,5	-1,37	3,0	1,0	0,63	42,04	2,21	13,43	
184	2011-01-04	2019-07-12	102	101	97	122,50	6,1	5	2	20,50	141,6	29,5	1,176	-0,044	-0,922	2,054	0,237	-0,941	168,0	1,36	3,0	1,0	0,20	6,12	2,83	10,66	
185	2011-01-04	2019-08-03	102	102	97	132,00	6,0	5	2	30,00	141,0	32,0	0,630	1,308	1,426	1,958	0,691	1,426	160,5	-0,11	2,5	1,0	0,20	10,05	1,34	23,55	
186	2011-03-16	2019-09-21	102	100	92	132,00	7,4	5	2	30,00	144,0	39,9	2,242	0,363	-1,134	2,428	1,812	-1,167	163,0	0,39	3,0	1,0	1,17	6,36	4,64	6,39	
187	2011-09-08	2020-03-20	102	101	99	132,00	7,0	4	2	30,00	135,3	30,0	1,107	0,397	-0,220	1,014	0,333	-0,243	152,5	-1,80	2,5	1,0	0,20	5,75	1,51	14,21	
188	2011-09-11	2020-04-10	102	101	101	120,00	5,5	3	1	18,00	123,1	22,3	-1,171	-1,446	-1,148	-1,266	-1,510	-1,165	155,0	-1,26	2,5	1,0	0,20	13,53	2,04	18,96	
189	2002-01-21	2010-09-02	103	102	96	132,00	6,1	4	2	29,00	131,4	28,0	0,876	0,691	0,376	0,234	-0,130	0,350	158,5	-0,52	3,0	1,0	2,37	43,94	3,13	16,17	
190	2002-07-22	2011-02-22	103	102	100	108,00	4,2	3	1	5,00	134,5	32,0	0,326	-0,066	-0,330	0,783	0,628	-0,352	161,0	-0,01	2,0	1,0	0,20	15,34	1,37	15,66	
191	2002-07-16	2011-02-25	103	101	98	122,50	4,1	2	1	19,50	126,2	26,0	-0,556	-0,449	-0,252	-0,743	-0,578	-0,297	161,0	-0,01	1,5	1,0	0,20	5,95	2,92	26,66	
192	2009-03-14	2017-10-28	103	100	88	132,00	6,1	5	2	29,00	134,0	32,0	0,974	0,814	0,487	0,696	0,628	0,409	155,0	-1,26	2,0	1,0	0,23	5,03	4,06	8,96	
193	2010-11-25	2019-06-28	103	100	97	127,25	4,5	2	1	24,25	134,9	27,3	1,130	-0,090	-0,960	0,853	-0,281	-1,014	166,5	1,07	1,5	1,0	0,20	9,33	3,14	11,52	
194	2011-08-02	2020-04-01	103	102	97	138,00	4,9	3	1	35,00	127,7	26,7	-0,361	-0,351	-0,250	-0,454	-0,415	-0,273	161,5	0,09	2,5	1,0	0,20	9,97	0,74	12,64	
195	2002-11-05	2011-07-05	104	101	99	132,00	4,4	3	1	28,00	135,4	33,0	1,124	0,920	0,539	0,847	0,733	0,461	159,5	-0,32	2,0	1,0	0,20	14,32	1,06	12,54	
196	2009-12-15	2018-09-08	104	103	97	108,00	4,5	3	1	4,00	134,3	29,5	0,749	0,174	-0,281	0,657	0,111	-0,304	157,0	-0,84	3,0	1,0	0,45	27,37	1,13	12,37	
197	2010-12-05	2019-08-16	104	99	96	122,50	4,3	3	1	18,50	134,0	29,0	0,585	1,301	1,442	0,605	0,013	1,301	157,5	-0,73	2,0	1,0	0,20	19,85	1,39	9,79	
198	2011-01-15	2019-09-28	104	102	94	114,00	3,7	2	1	10,00	131,3	34,7	0,789	0,139	-0,364	0,124	0,999	-0,408	158,5	-0,52	3,0	1,0	0,20	8,25	3,49	19,58	
199	2011-02-12	2019-10-17	104	102	98	122,50	4,3	2	1	18,50	129,8	22,8	0,033	-1,360	-1,928	-0,151	-1,487	-1,948	163,0	0,39	3,0	1,0	0,20	6,44	3,41	16,14	
200	2011-06-26	2020-03-11	104	103	97	126,00	5,1	2	1	22,00	133,7	26,0	0,644	-0,578	-1,285	0,552	-0,642	-1,301	153,5	-1,58	2,0	1,0	0,20	41,91	1,34	13,45	
201	2011-06-16	2020-03-14	104	103	100	120,00	1,5	2	1	16,00	134,1	31,3	0,714	0,507	0,222	0,622	0,445	0,198	162,5	0,29	2,5	1,0	0,20	29,87	2,21	17,02	
202	2011-08-26	2020-05-22	104	100	94	120,00	6,1	4	2	16,00	135,8	29,5	1,285	0,363	-0,401	0,915	0,111	-0,487	165,5	0,88	3,0	1,0	0,20	18,14	0,99	9,66	
203	2011-10-04	2020-07-01	104	104	104	120,00	4,0	2	1	16,00	129,4	30,8	-0,225	0,355	0,628	-0,225	0,355	0,628	162,4	0,27	1,0	1,0	0,20	8,49	2,40	6,29	
204	2011-10-17	2020-07-03	104	103	101	120,00	5,1	4	1	16,00	136,3	44,4	1,092	2,247	2,516	1,000	2,192	2,489	167,0	1,17	2,0	1,0	2,19	27,34	3,19	10,14	
205	2003-01-21	2011-11-11	105	102	98	144,00	4,6	3	1	39,00	131,7	30,5	1,014	1,179	0,997	0,105	0,239	0,916	156,0	-1,05	2,0	1,0	0,66	42,29	1,95	8,56	
206	2010-12-07	2019-09-07	105	102	96	108,00	5,6	3	1	3,00	135,3	35,1	-0,285	0,691	1,123	0,737	0,999	1,042	159,0	-0,42	3,0	1,0	0,21	19,82	2,84	12,85	
207	2010-12-23	2019-09-27	105	104	102	115,25	3,5	2	1	10,25	128,1	32,0	0,196	0,300	0,279	-0,560	0,506	0,254	161,5	0,09	2,5	1,0	0,20	5,05	1,23	9,68	
208	2011-01-16	2019-10-16	105	104	100	132,00	5,8	4	1	27,00	138,8	45,1	1,417	2,262	2,352	1,323	2,207	2,325	159,0	-0,42	2,5	1,0	0,72	15,43	4,22	9,97	
209	2011-01-15	2019-10-29	105	104	104	122,50	4,1	3	1	17,50	128,9	22,3	-0,319	-1,636	-2,024	-0,410	-1,698	-2,033	166,0	0,98	2,0	1,0	0,20	5,36	3,04	15,26	
210	2011-03-30	2020-01-28	105	102	98	122,50	5,0	4	1	17,50	130,0	28,8	0,070	0,099	0,077	-0,205	-0,090	0,006	163,5	0,49	3,0	1,0	0,20	12,08	2,51	16,92	
211	2011-04-16	2020-02-13	105	105	101	127,25	4,3	3	1	22,25	132,6	29,0	0,266	-0,049	-0,257	0,266	-0,049	-0,257	163,5	0,49	2,5	1,0	0,20	14,14	1,90	16,73	
212	2011-06-08	2020-03-10	105	102	101	122,50	5,5	3	1	17,50	131,1	24,4	0,272	-0,915	-1,487	-0,004	-1,104	-1,529	159,0	-0,42	2,0	1,0	0,20	17,40	1,91	14,67	
213	2011-09-03	2020-06-24	105	104	101	126,00	4,6	3	1	21,00	127,8	27,9	-0,527	-0,214	0,049	-0,617	-0,276	0,025	154,5	-1,37	3,0	1,0	0,20	11,97	0,73	6,17	
214	2011-10-17	2020-07-17	105	102	99	120,00	5,4	4	1	15,00	128,5	29,3	-0,209	0,198	0,400	-0,485	0,010	0,325	155,5	-1,15	3,0	1,0	0,20	20,81	0,89	16,21	
215	2002-10-02	2011-08-04	106	102	96	132,00	4,5	3	1	26,00	131,8	30,5	0,736	0,936	0,824	0,032	0,178	0,718	159,5	-0,32	1,5	1,0	1,13	23,63	3,39	16,51	
216	2004-08-27	2013-07-02	106	105	103	132,00	5,9	3	1	26,00	133,7	33,5	0,123	0,239	0,242	0,370	0,694	0,218	161,0	-0,01	2,5	1,0	0,29	16,63	0,72	12,30	
217	2010-10-22	2019-08-30	106	106	102	115,25	5,1	3	1	9,25	126,7	23,0	-0,920	-1,551	-1,462	-0,920	-1,551	-1,462	151,0	-2,14	2,5	1,0	0,20	8,64	2,80	13,29	
218	2010-12-01	2019-10-22	106	105	101	122,50	4,2	2	1	16,50	134,8	35,2	0,651	1,013	0,992	0,561	0,954	0,966	160,0	-0,21	3,0	1,0	0,20	12,54	2,79	20,19	
219	2011-03-31	2020-02-10	106	105	102	114,00	5,8	4	1	8,00	136,4	30,3	0,924	0,202	-0,360	0,834	0,141	-0,381	159,0	-0,42	2,0	1,0	0,20	9,39	1,59	14,72	
220	2011-05-31	2020-04-02	106	105	103	122,50	4,1	3	1	16,50	134,2	27,3	0,547	-0,406	-0,959	0,457	-0,467	-0,977	162,5	0,29	3,0	1,0	0,50	13,40	3,30	13,10	
221	2011-07-02	2020-05-12	106	105	105	120,00	4,8	3	1	14,00	130,0	29,4	-0,205	0,030	0,170	-0,296	-0,031	0,146	157,8	-0,67	2,0	1,0	0,20	8,07	1,28	16,00	
222	2002-01-29	2011-01-06	107	105	101	132,00	4,7	3	1	25,00	135,4	35,5	0,460	0,419	0,271	0,575	0,941	0,222	160,5	-0,11	2,0	1,0	0,20	14,94	1,88	12,46	
223	2002-03-06	2011-02-12	107	106	100	108,00	6,6	3	2	1,00	133,7	31,5	0,732	0,270	-0,126	0,280	0,299	-0,148	161,0	-0,01	2,0	1,0	0,28	20,58	3,12	20,18	
224	2002-03-06	2011-02-12	107	106	90	108,00	5,5	3	1	1,00	135,8	31,0	-0,595	-1,409	-1,507	0,643	0,210	-1,520	161,0	-0,01	2,0	1,0	0,39	53,67	1,30	11,02	
225	2002-02-21	2011-02-19	107	106	104	108,00	5,6	4	1	1,00	128,4	23,5	0,664	0,998	0,963	-0,686	-1,469	0,936	155,0	-1,26	2,0	1,0	0,84	25,91	2,57	10,53	
226	2002-07-11	2011-07-09	107	107	102	132,00	5,2	3	1	25,00	137,0	30,0	0,845	0,025	-0,555	0,845	0,025	-0,555	163,5	0,49	3,0	1,0	1,25	41,99	2,26	11,37	
227	2010-07-01	2019-06-19	107	106	103	122,50	5,1	3	1	15,50	141,3	30,4	0,868	1,210	1,137	1,544	0,100	1,111	169,5	1,64	3,0	1,0	0,32	17,26	2,40	13,58	
228	2010-08-26	2019-08-22	107	107	105	138,00	6,1	4	2	31,00	138,9	37,8	1,159	1,260	1,023	1,159	1,260	1,023	165,0	0,78	2,0	1,0	0,36	34,33	2,33	17,97	
229	2010-09-12	2019-08-22	107	106	105	127,25	4,3	3	1	20,25	132,9	35,5	1,634	0,160	-0,939	0,139	0,941	-0,957	156,9	-0,86	2,5	1,0	0,20	12,34	1,96	20,39	
230	2010-10-13	2019-09-29	107	106	103	122,50	5,1	4	1	15,50	136,6	37,0	0,229	0,998	1,236	0,778	1,153	1,209	161,5	0,09	2,5	1,0	0,20	7,89	2,18	12,44	
231	2010-10-09	2019-10-08	107	107	106	132,00	5,9	3	1	25,00	134,7	28,7	0,454	-0,231	-0,640	0,454	-0,231	-0,640	168,4	1,44	2,0	1,0	0,51	9,03	2,39	6,79	
232	2011-01-24	2019-12-26	107	106	102	127,25	4,8	3	1	20,25	136,7	29,1	0,885	-0,090	-0,751	0,795	-0,150	-0,769	164,0	0,59	2,0	1,0	0,47	25,75	1,09	8,17	
233	2011-01-19	2020-01-18	107	106	98	150,50	6,2	5	2	43,50	140,0	31,7	1,427	0,394	-0,438	1,337	0,334	-0,459	160,0	-0,21	3,5	2,0	2,78	75,69	4,14	14,12	
234	2011-04-06	2020-03-21	107	105	99	132,00	4,3	2	1	25,00	130,8	26,1	-0,058	-0,680	-0,905	-0,239	-0,802	-0,941	160,8	-0,05	3,0	1,0	0,20	24,88	3,59	11,67	
235	2011-07-06	2020-06-08	107	105	102	126,00	4,9	2	1	19,00	132,4	33,7	0,230	0,785	0,936	0,050	0,667	0,883	164,5	0,69	2,5	1,0	0,20	7,97	1,86	8,97	
236	2011-06-20	2020-06-16	107	106	99	120,00	3,2	2	1	13,00	127,9	24,7	-0,690	-1,087	-1,010	-0,781	-1,147	-1,027	159,5	-0,32	2,0	1,0	0,20	11,59	1,11	15,80	
237	2002-03-11	2011-03-12	108	107	103	108,00	5,2	3	1	0,00	129,0	31,0	1,240	-0,376	-1,426	-0,667	0,147	-1,443	168,5	1,45	2,5	1,0	0,20	8,74	1,74	12,37	
238	2010-09-10	2019-09-26	108	107	103	122,50	4,2	3	1	14,50	135,4	36,6	0,575	1,097	1,160	0,482	1,037	1,133	162,5	0,29	2,0	1,0	0,30	15,96	3,28	12,92	
239	2010-09-25	2019-10-08	108	108	102	132,00	4,3	3	1	24,00	137,6	38,4	0,853	1,279	1,247	0,853	1,279	1,247	162,0	0,19	3,0	1,0	0,20	8,95	1,49	9,00	
240	2010-10-06	2019-10-16	108	106	106	122,50	4,8	3	1	14,50	138,6	32,3	1,200	0,497	-0,124	1,019	0,375	-0,170	163,5	0,49	2,0	1,0	0,20	5,54	3,76	15,11	
241	2010-09-27	2019-10-18	108	107	94	138,00	6,2	5	2	30,00	134,9	30,6	0,489	0,137	-0,146	0,396	0,074	-0,170	160,5	-0,11	3,0	1,0	0,20	44,39	1,69	13,70	
242	2010-10-01	2019-10-26	108	107	101	132,00	5,3	4	1	24,00	135,0	34,5	0,506	0,791	0,768	0,413	0,729	0,741	159,5	-0,32	2,5	1,0	0,20	9,59	2,05	9,65	
243	2010-10-28	2019-11-11	108	107	101	127,25	2,7	2	1	19,25	128,4	26,7	-0,686	-0,662	-0,448	-0,780	-0,726	-0,470	155,0	-1,26	3,0	1,0	0,20	7,26	2,93	14,20	
244	2010-11-23	2019-11-29	108	107	105	115,25	3,8	3	1	7,25	128,6	32,0	-0,648	0,386	0,931	-0,742	0,324	0,904	160,5	-0,11	2,5	1,0	0,20	5,12	1,63	11,84	
245	2011-01-07	2020-01-09	108	103	97	122,50	5,7	4	1	14,50	140,4	35,7	1,769	1,205	0,534	1,310	0,909	0,408	158,5	-0,52	2,0	1,0	2,90	7,20	3,00	20,80	
246	2011-01-26	2020-02-15	108	107	92	132,00	5,1	4	1	24,00	140,6	36,1	1,433	1,027	0,497	1,342	0,966	0,471	159,5	-0,32	4,0	2,0	2,86	103,31	5,63	26,03	
247	2011-03-25	2020-04-03	108	106	101	126,00	6,0	5	2	18,00	135,0	28,6	0,595	-0,191	-0,687	0,413	-0,315	-0,727	163,5	0,49	2,5	1,0	0,20	8,83	1,31	14,05	
248	2008-07-02	2017-08-05	109	107	99	144,00	5,1	3	1	35,00	137,9	36,6	0,995	1,097	0,891	0,810	0,977	0,839	158,5	-0,52	3,0	1,0	2,06	24,58	3,95	13,02	
249	2010-02-13	2019-04-05	109	107	101	122,50	6,4	4	2	13,50	134,3	36,7	0,385	1,111	1,298	0,199	0,991	1,244	156,0	-1,05	3,0	1,0	2,01	50,86	4,28	21,37	
250	2001-10-20	2011-02-18	111	107	98	132,00	4,3	3	1	21,00	129,9	27,0	-0,405	-0,594	-0,546	-0,773	-0,843	-0,632	158,0	-0,63	2,0	1,0	0,33	48,87	2,41	22,06	
251	2002-02-27	2011-06-11	111	105	97	132,00	5,7	3	1	21,00	139,4	33,0	1,420	0,673	-0,022	0,873	0,309	-0,162	161,0	-0,01	3,0	1,0	4,21	34,35	3,31	12,62	
252	2006-10-05	2016-01-08	111	110	104	132,00	6,0	4	2	21,00	134,8	299,0	0,193	7,492	10,733	0,101	7,535	10,850	164,5	0,69	3,0	1,0	0,30	39,63	1,75	9,97	
253	2001-09-04	2011-01-26	112	106	101	108,00	9,0	5	2	-4,00	131,3	27,0	-0,058	-0,534	-0,703	-0,609	-0,903	-0,824	155,0	-1,26	2,0	1,0	0,77	52,48	7,57	24,34	
254	2002-05-02	2011-09-21	112	112	102	132,00	6,6	4	2	20,00	141,1	43,0	1,057	1,605	1,586	1,057	1,605	1,586	157,0	-0,84	4,0	3,0	11,00	108,71	4,47	15,55	
255	2008-04-22	2017-09-01	112	111	106	144,00	7,8	5	2	32,00	147,3	43,8	2,095	1,747	1,134	2,009	1,693	1,107	161,0	-0,01	3,5	1,0	0,84	66,17	2,77	11,31	
256	2001-09-27	2011-05-09	115	114	90	108,00	4,7	3	1	-7,00	139,5	38,5	0,615	0,945	0,911	0,522	0,885	0,885	157,5	-0,73	4,0	1,0	0,49	37,81	3,91	18,82	
257	2004-01-28	2013-09-24	115	115	115	138,00	4,1	2	1	23,00	134,6	26,0	-0,301	-1,309	-1,586	-0,301	-1,309	-1,586	161,5	0,09	2,0	1,0	0,10	33,00	3,00	12,90	
258	2003-06-14	2013-02-14	116	115	103	144,00	7,8	4	2	28,00	149,0	40,0	1,996	1,079	0,197	1,909	1,021	0,173	160,5	-0,11	5,0	3,0	3,79	71,74	1,70	61,56	
259	2001-04-26	2011-02-15	117	114	96	122,50	6,7	5	2	5,50	130,9	27,0	-0,863	-1,022	-0,815	-1,136	-1,196	-0,873	157,5	-0,73	3,5	1,0	0,34	31,09	2,81	15,68	
260	2001-09-09	2011-07-28	118	114	96	132,00	4,5	3	1	14,00	131,5	27,0	-0,755	-1,022	-0,888	-1,118	-1,253	-0,963	155,5	-1,15	2,0	1,0	3,03	21,40	3,25	8,71	
261	2000-07-06	2011-07-12	132	132	108	132,00	5,6	3	1	0,00	139,0	33,0	-1,063	-0,910	-0,570	-1,063	-0,910	-0,570	156,0	-1,05	4,0	3,0	2,07	49,92	6,08	19,29	
